# Comment on ‘A comparison of three methods for detecting KRAS mutations in formalin-fixed colorectal cancer specimens’

**DOI:** 10.1038/bjc.2012.431

**Published:** 2012-09-25

**Authors:** N Normanno, A Rachiglio, C Roma, F Ciardiello, C Pinto

**Affiliations:** 1 Cell Biology and Biotherapy Unit, INT-Fondazione Pascale, Naples 80131, Italy; 2 Laboratory of Pharmacogenomic, Centro di Ricerche Oncologiche di Mercogliano–CROM Mercogliano (AV) 83013, Italy; 3 Medical Oncology, Department of Experimental and Clinical Medicine and Surgery F. Magrassi and A. Lanzara, Second University of Naples, Naples, Italy; 4 Medical Oncology, S Orsola-Malpighi Hospital, Bologna, Italy

**Sir**,

The study by Gonzalez de Castro *et al* (2012) published in *British Journal of Cancer* compared the sensitivity and specificity of three different methods, the COBAS KRAS mutation kit by Roche (Basel, Switzerland), the Therascreen KRAS kit by Qiagen (Hilden, Germany) and direct Sanger sequencing of PCR product (PCR/sequencing), to detect KRAS mutations in formalin-fixed paraffin-embedded tissues from colorectal carcinoma (CRC) patients. The study clearly demonstrated the good reproducibility of the COBAS test. However, we feel that some conclusions might be misleading.

The authors compared COBAS and PCR/sequencing in a cohort of samples including specimens with high levels of necrosis, low tumour content and low-frequency mutation. The COBAS showed higher sensitivity as compared with PCR/sequencing, although a surprising excellent agreement between the two methods was found in the most challenging specimens. However, the sensitivity of PCR/sequencing can be significantly improved by enrichment of the tumour cell content through macrodissection, which was not performed in this study. Therefore, we feel that the results obtained by the authors might be flawed by the inappropriate processing of the specimens. In this regard, we and other groups have previously demonstrated that real-time PCR-based techniques such as the Therascreen kit are superior to PCR/sequencing only in specimens with ⩽30% tumour cells after macrodissection, which are relatively rare in CRC (Carotenuto *et al*, 2010; Tol *et al*, 2010).

The authors stressed in their conclusions the importance to assess all KRAS mutations, including codon 61 mutations, in agreement with the approval by the European Medical Agency of anti-EGFR monoclonal antibodies (MAbs) for KRAS wild-type CRC patients. However, the clinical studies that led to the approval of EGFR MAbs for KRAS wild-type CRC patients only investigated codons 12 and 13 mutations, and in the majority of the studies only the seven most frequent KRAS mutations detected by the Therascreen kit were assessed (Amado *et al*, 2008; Karapetis *et al*, 2008; Van Cutsem *et al*, 2009; Bokemeyer *et al*, 2011). The data regarding the role of codon 61 mutations in the resistance to anti-EGFR agents in CRC have not been obtained in the context of randomized clinical trials. In addition, these data are related only to patients that have been treated with anti-EGFR MAbs as monotherapy in third or further lines of therapy, or that received cetuximab to revert resistance to irinotecan (De Roock *et al*, 2011). These findings cannot be transferred to patients treated in first or second line with combinations of polychemotherapy regimens and anti-EGFR MAbs, as recently suggested for BRAF mutations (Van Cutsem *et al*, 2011). Therefore, the correct interpretation of these mutations is that their role in the resistance to anti-EGFR MAbs in CRC has not been proven yet.

Analysis with COBAS resulted in two false-positive cases (one in each site). We believe the fact that this system does not allow the operator to analyse the amplification curves and provides only a result of ‘mutation detected’ or ‘mutation not detected’, might lead to misleading results that could be avoided by the analysis of raw data by experienced molecular biologists.

The COBAS kit does not distinguish between mutations in codons 12 and 13. Although the role of G13D mutation in the resistance to anti-EGFR MAbs is not clear because of the contrasting results that have been reported up to now (Peeters *et al*, 2011; Tejpar *et al*, 2012), the fact that the COBAS KRAS kit does not tell between codons 12 and 13 mutations limits the possibility to increase our knowledge on the outcome of these different mutations.

In conclusion, the superiority of COBAS and more generally of real-time PCR-based methods over PCR/sequencing is limited to a small fraction of CRC specimens, and reporting of rare KRAS mutations not investigated in randomized clinical trials should be accompanied by a cautious interpretation.
